# Survey of Freshly Harvested Oat Grains from Southern Brazil Reveals High Incidence of Type B Trichothecenes and Associated *Fusarium* Species

**DOI:** 10.3390/toxins13120855

**Published:** 2021-12-01

**Authors:** Mariana Pinheiro, Caio H. T. Iwase, Bruno G. Bertozzi, Elem T. S. Caramês, Lorena Carnielli-Queiroz, Nádia C. Langaro, Eliana B. Furlong, Benedito Correa, Liliana O. Rocha

**Affiliations:** 1 Department of Food Science and Nutrition, Food Engineering Faculty, State University of Campinas—UNICAMP, Campinas 13083-862, Brazil; marypinhe163@gmail.com (M.P.); caioiwase@yahoo.com.br (C.H.T.I.); bgbertozzi@gmail.com (B.G.B.); elem.carames@gmail.com (E.T.S.C.); 2 Institute of Biomedical Sciences, University of Sao Paulo, São Paulo 05508-000, Brazil; lcarnielliqueiroz@gmail.com (L.C.-Q.); correabe@usp.br (B.C.); 3 Faculty of Agronomy and Veterinary Medicine, University of Passo Fundo, Passo Fundo 99042-800, Brazil; nclangaro@upf.br; 4 School of Chemistry and Food, Federal University of Rio Grande, Rio Grande 96203-900, Brazil; dqmebf@furg.br

**Keywords:** oats, *Fusarium sambucinum* species complex, deoxynivalenol, nivalenol, mycotoxin

## Abstract

The current study investigated the fungal diversity in freshly harvested oat samples from the two largest production regions in Brazil, Paraná (PR) and Rio Grande do Sul (RS), focusing primarily on the *Fusarium* genus and the presence of type B trichothecenes. The majority of the isolates belonged to the *Fusarium sambucinum* species complex, and were identified as *F. graminearum* sensu stricto (s.s.), *F. meridionale*, and *F. poae*. In the RS region, *F. poae* was the most frequent fungus, while *F. graminearum* s.s. was the most frequent in the PR region. The *F. graminearum* s.s. isolates were 15-ADON genotype, while *F. meridionale* and *F. poae* were NIV genotype. Mycotoxin analysis revealed that 92% and 100% of the samples from PR and RS were contaminated with type B trichothecenes, respectively. Oat grains from PR were predominantly contaminated with DON, whereas NIV was predominant in oats from RS. Twenty-four percent of the samples were contaminated with DON at levels higher than Brazilian regulations. Co-contamination of DON, its derivatives, and NIV was observed in 84% and 57.7% of the samples from PR and RS, respectively. The results provide new information on *Fusarium* contamination in Brazilian oats, highlighting the importance of further studies on mycotoxins.

## 1. Introduction

Oats (*Avena sativa* L.) have been consumed by humans and livestock since ancient times; it is considered a nutrient-rich cereal due to the high concentration of lipids, proteins, vitamins, antioxidants, minerals, and β-glucan [1]. The global production of oats in 2021/2021 was 25,570 thousand metric tons, with the European Union being the largest producer followed closely by Canada, Russia, Australia, United Kingdom and Brazil [2].

Over the last five years, oat production has increased in Brazil, with a reported grain productivity of 2426 kg/hectare and production of 1087.1 thousand tons [3]. However, it is important to highlight that over the last seasons, heavy rain conditions were reported in the southern region, where most of the small-grain cereals, such as oats, are produced. The increased humidity potentially favored fungal infections and reduced grain quality [3].

Cereals can be affected by fungal diseases, which can lead to lower nutritional values and mycotoxin accumulation in the grains, resulting in reduced product quality and economic losses [4]. Numerous fungi may be attributed to various oat diseases; the *Fusarium* genus, however, is considered one of the major threats. One of the most serious and economically important diseases caused by the genus is Fusarium head blight (FHB), which affects cereal production worldwide [5,6,7].

Fusarium head blight is primarily caused by species in the *Fusarium graminearum* species complex (FGSC); however, other *Fusarium* species may also be involved. FHB causes flower abortion and the formation of pitted, wrinkled, and rough grains that are “pinkish” in color [8]. Infection by these pathogens can also result in mycotoxin accumulation, mainly trichothecenes and zearalenone (ZEN) [9,10].

Trichothecenes produced by *Fusarium* species are classified into either type A or B; these compounds are differentiated by the C-8 function of the 12,13-epoxytrichothec-9-ene (EPT) core structure [11]. Members of the FGSC are able to produce type B trichothecenes such as deoxynivalenol (DON) and its acetylated derivatives (3 acetyl-DON and 15 acetyl-DON; 3-ADON and 15-ADON) as well as nivalenol (NIV) and its acetylated forms [11].

In animals, DON has been linked to feed refusal, vomiting, and weight reduction [12]. NIV can cause immunotoxicity and hematotoxicity, based on in vitro and in vivo tests [13]. The toxic effects of the acetylated DON forms are poorly documented; however, 15-ADON has been reported to be more toxic than DON and 3-ADON in ex vivo and in vivo tests using human intestinal cells and piglets [14]. Due to the toxic effects of DON in humans, a provisional maximum tolerable daily intake (PMTDI) of 1.0 μg/kg body weight/day has been set by the UN Food and Agriculture Organization/World Health Organization Joint Expert Committee on Food Additives (JECFA) [15]. For NIV, a tolerable daily intake (TDI) of 1.2 μg/kg body weight/day has been set by the European Food Safety Authority (EFSA) [13].

Zearalenone is a cyclic compound containing a resorcyclic acid lactone structure, and it is also primarily produced by the same fungi that produce type B trichothecenes. It is commonly found together with DON and NIV in cereals. ZEN is considered an estrogenic mycotoxin that causes abnormalities in the reproductive system, particularly in swine, leading to infertility, genital prolapse, and enlarged mammary glands [16]. Due to the fact of these effects, the JECFA established a PMTDI of 0.5 μg/kg body weight/day [17].

In the Northern Hemisphere, the main *Fusarium* species associated with oats are *F. graminearum*, *F. avenaceum*, *F. sporotrichioides*, *F. langsethiae*, *F. poae*, *F. culmorum*, and *F. tricinctum* [5,6,7,18,19,20]. This implies that a diverse range of mycotoxins may be found in oats. For example, *F. sporotrichioides* and *F. langsethiae* are responsible for T-2 and HT-2 (type A trichothecenes) accumulation in small grain cereals [21]; whereas *F. graminearum* and *F. culmorum* are able to produce ZEN and type B trichothecenes; *F. poae* mainly produces NIV [22]; finally, *F. avenaceum* and *F. tricinctum* are able to produce other *Fusarium* mycotoxins such as moniliformin (MON) and enniatins (ENNs) [23]. Indeed, several studies have already shown the occurrence of type A and type B trichothecenes in oats grown in colder climates [7,24,25,26,27,28].

In South America, a few studies have shown that *Alternaria*, *Aspergillus*, *Penicillium,* and *Fusarium* are prevalent in oats [29,30,31]. Regarding the *Fusarium* genus, *F. graminearum*, *F. poae*, and *F. verticillioides* have previously been recovered from freshly harvested grains [31]. In regard to mycotoxin contamination, aflatoxin B1 (AFB1), DON, fumonisin B1 (FB1), and ochratoxin A (OTA) have been detected in oat grains and products [29,31,32,33,34,35].

In Brazil, reports of fungi and mycotoxin contamination in oats are scarce, with a few only focusing either on the mycobiota or mycotoxin contamination. The majority of the associated fungi recovered were *Alternaria*, *Drechslera*, *Fusarium*, and *Puccinia* [29,30,36]. However, the only reported mycotoxin was FB1, while aflatoxins, ochratoxin A, and ZEN were not detected [29,33].

Due to the lack of information on fungal diversity and mycotoxin contamination in Brazilian oats, together with the increasing production of this cereal within the country, the objectives of the current study were to (i) characterize *Fusarium* species associated with freshly harvested oats, recovered from the largest production regions of Brazil; (ii) determine the levels of deoxynivalenol, its derivatives (3-acetildeoxynivalenol and 15-acetildeoxynivalenol), and nivalenol in the grain samples.

## 2. Results

### 2.1. Water Activity and Mycobiota of Freshly Harvested Oat Grains

Ninety-two percent of the oat samples were contaminated with fungi, predominantly by the *Fusarium* genus. In the RS region, samples were contaminated with *Fusarium*, followed by *Phoma*, *Epicoccum*, *Alternaria*, *Cladosporium*, *Penicillium*, *Aspergillus*, *Drechslera*, *Pestalotiopsis*, *Mucor*, *Rhizopus*, *Curvularia*, and *Trichoderma*. Water activity (a_w_) ranged from 0.4 to 0.6 (mean = 0.54). No correlation between a_w_ and the occurrence of *Fusarium* was observed (*p* < 0.05). In the PR region, 33.1% of the samples were contaminated with *Fusarium*, followed by *Alternaria*, *Nigrospora*, *Epicoccum*, *Phoma*, *Cladosporium*, *Rhizopus*, *Penicillium*, *Drechslera*, *Mucor,* and *Pestalotiopsis* (Table 1). Water activity ranged from 0.5 to 0.6 (mean = 0.51). No correlation between a_w_ and the occurrence of *Fusarium* was observed (*p* < 0.05).

The *F. sambucinum* species complex (FSAMSC) was foremost in oat samples from both regions. In the RS region, *F. poae* was the primary species isolated, followed by the FGSC, *F. avenaceum*, and *F. proliferatum*. In the PR region, the majority of the isolates belonged to the FGSC, followed by *F. poae*, *F. incarnatum-equiseti* species complex (FIESC), *F. verticillioides*, *F. subglutinans*, and *F. solani* species complex.

### 2.2. Molecular Characterization of Fusarium Isolates

A subsample of *Fusarium* isolates were selected for molecular characterization. Isolates belonging to the FSAMSC were randomly selected for phylogenetic analysis of the second major subunit of the RNA polymerase locus (*RPB2*) and genotype characterization.

**Figure 1:**
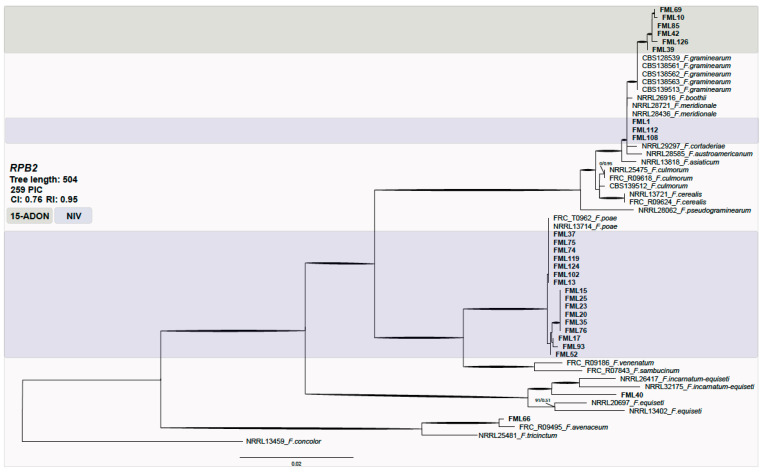
Maximum parsimony phylogeny inferred from the first fragment of the *RPB2* locus. Bootstrap values above 70% and Bayesian posterior probabilities (BPPs) above 0.9 are assigned in bold branches. Support values above branches are bootstrap/BPP values. The outgroup is *F. concolor*. The NIV genotype is highlighted in blue and 15-ADON in green. FML: *Food Microbiology Laboratory*.

The phylogenetic analysis data set consisted of 55 taxa with 259 parsimony informative characters (PICs). The analysis resulted in one hundred of the most parsimonious trees (consistency index (CI) = 0.76; retention index (RI) = 0.95). No significant topological variations were detected between neighbor-joining, parsimony, and likelihood phylogenies (data not shown). Most of the isolates were clustered within *F. graminearum* and *F. poae* species and a few within *F. meridionale* (Figure 1).

The FGSC isolates were predominantly of the 15-ADON (50%) genotype, followed by NIV (36.4%) and 3-ADON (13.6%). All strains identified as *F. meridionale* were of the NIV genotype. All *F. graminearum* s.s. were characterized as 15-ADON, while *F. austroamericanum* were of the 3-ADON genotype. As expected, all of the *F. poae* strains displayed the NIV genotype. The oat grain isolates from Rio Grande do Sul were identified mostly as of the NIV (75%) genotype, followed by 15-ADON (16.7%) and 3-ADON (8.3%). The isolates from Paraná mostly displayed the NIV genotype (70.4%), followed by 15-ADON (25.9%) and 3-ADON (3.7%).

### 2.3. Mycotoxin Analysis

#### Occurrence of Type B Trichothecenes

Type B trichothecenes were found in 92% and 100% of oat samples from PR and RS, respectively. In PR, DON was the predominant mycotoxin and was detected in 44.2% of the samples, followed by NIV (28.6%), 3-ADON (18.8%), and 15-ADON (7.7%). In RS, NIV was detected in 44.7% of the samples and was the predominant mycotoxin, followed by DON (35%), 15-ADON (3.6%), and 3-ADON (14.8%).

Table 2 shows the levels of DON, 3-ADON, 15-ADON, and NIV detected in the PR and RS regions. The mean contamination levels for mycotoxins in oat samples from PR were 45, 18.8, 7.7, and 28.6 µg/kg for DON, 3-ADON, 15-ADON, and NIV, respectively. Regarding the RS oat samples, the mean mycotoxin contamination levels were 35, 14.8, 3.6, and 46.7 µg/kg for DON, 3-ADON, 15-ADON, and NIV, respectively.

In the current study, a higher frequency and average concentration levels of NIV were found in RS; however, no significant difference was observed between RS and PR for the other evaluated mycotoxins (*p* > 0.05).

The co-occurrence of trichothecenes was also observed. Eighty-four percent of the samples from the PR region simultaneously presented DON and NIV, whereas the co-occurrence of DON and NIV was observed in only 57.7% of the RS samples.

## 3. Discussion

The current study found a high diversity of fungi in Brazilian oat grains including potentially toxigenic fungi. The occurrence of the FSAMSC and its related mycotoxins, such as DON, 3-ADON, 15-ADON, and NIV, was the focus of the investigation. It is important to mention that there is a lack of research regarding mycotoxin contamination in Brazilian oat grains, despite high consumption by the population. Most of the previous studies were conducted in the Northern Hemisphere, and they have reported the presence of multiple *Fusarium* mycotoxins in oat grains [37,38].

A study conducted on Swiss oat samples from the 2013 to 2015 harvests reported the occurrence of nine different *Fusarium* species and a 97% frequency of *Fusarium* infection in the analyzed samples; similar to the frequency determined in this study (93.8% and 85.5% for samples from RS and PR, respectively). The same study pointed out that *F. poae* was the most predominant species in all three harvest years (2013, 2014 and 2015) with 55%, 57% and 87% isolation amongst *Fusarium* species [7].

In the current study, most of *Fusarium* isolates belonged to the FSAMSC and were characterized as *F. graminearum* s.s., *F. meridionale* and *F. poae*. The latter was frequently isolated from RS samples, in contrast to the PR samples, where *F. meridionale* was highly detected. Studies have highlighted *F. poae* as a frequent species found in oat samples [7,39]. These results suggest that different geographic origins, soil type, and environmental and harvest conditions could lead to a distinct predominant species and might influence the mycotoxin content of the grains [40].

In both studied regions, the NIV genotype was predominant. In RS, it was associated with the high frequency of *F. poae* and with the samples mostly contaminated with the NIV mycotoxin. The high occurrence of this genotype in PR was associated to *F. meridionale* and *F. poae*. This knowledge is relevant for determining a more efficient prediction of the contamination by NIV, and it may aid management strategies to control the occurrence of toxigenic fungi in barley from different geographic regions [41].

Mycotoxin analysis demonstrated that most of the samples were contaminated with type B trichothecenes. DON contamination was higher in samples from PR, while NIV was prevalent in RS. The presence of these mycotoxins conformed with the frequency of isolated fungi, as *F. poae* was the most isolated species in RS and *F. graminearum* s.s. in PR. It has been reported that the incidence of *F. poae* increases when the climatic conditions do not favor the proliferation of *F. graminearum* s.s., the dominant pathogen involved in FHB [42,43].

In Brazil, previous studies revealed a high frequency of the FSAMSC in wheat, barley, and rice, leading to the high occurrence of DON in grains. Despite this knowledge, information correlating mycotoxin contamination to the predominant species in Brazilian oats is still scarce [44,45,46,47,48,49,50,51]. In Europe, the high occurrence of *F. poae* in cereals is responsible for NIV contamination [37,52], while in Asia, NIV contamination is attributed to *F. asiaticum* [53,54]. In South America, NIV was found in wheat from Argentina and Brazil in lower frequency and levels than DON. Apparently, the higher frequency of DON is related to the higher risk of FHB epidemics caused by the predominance of no-till cropping and climate change in the subtropical environment of Southern Brazil [45,55]. Furthermore, the analysis detected the presence of 3-ADON and 15-ADON in oats, with high levels of 3-ADON in samples from both regions studied. This result corroborates with the occurrence of the FGSC 3-ADON genotype. In Europe, the acetylated DON forms are reported in cereals such as oats [52,56,57,58], maize, and wheat [59].

In our study, 24% of the samples presented DON levels higher than the maximum limit established by Brazilian legislation (750 µg/kg). Despite the absence of legislation for NIV globally, this mycotoxin was present in high levels, mainly in samples from RS. The toxic effects of NIV are still inconclusive, although it has displayed immunotoxic and hematotoxic effects, which can be critical to humans [13]. In the case of acetylated DON forms (i.e., 15-ADON and 3-ADON), high levels of 3-ADON were observed in the grains from both regions. Information about its toxic effects in animals and humans are still scarce, but a study demonstrated that 15-ADON was more toxic than DON and 3-ADON [14].

Co-contamination of DON, 15-ADON, 3-ADON, and NIV was observed in this study due to the presence of different fungi in the grains. To our knowledge, this is the first report demonstrating the co-occurrence of these mycotoxins in Brazilian oat grains and their correlation with associated *Fusarium* species. However, the co-occurrence of DON and NIV has already been reported in 86% of Brazilian wheat kernels analyzed [45] as well as in 29.6% of Brazilian barley samples [51]. The main concerns about co-contamination are the possible interactions and potential synergistic effects that these mycotoxins may have on animal and human health. Cheat et al. (2015) [60] reported that the toxic effects of DON were intensified when consumed with NIV in in vitro models.

Overall, studies about mycotoxin contamination in oat grains are relevant and necessary to determine an efficient risk control plan, as the consumption of oats in natura plant-based beverages or cereal-based foods has been increasing, boosted mainly by its good nutritional features such as high protein and dietary fiber content [61].

Since the levels of mycotoxin contamination and the dominant species in cereals can change according to various environmental parameters, studies that elucidate the prevalence of toxigenic fungi in different geographic regions are vital for designing efficient control management strategies, aiding producers in obtaining a safer product. The results of this study highlighted the importance of further research on the contamination of multiple *Fusarium* mycotoxins in oat grains and their by-products consumed in Brazil.

## 4. Conclusions

This study showed high recovery of *F. graminearum* s.s. and *F. poae* from Brazilian oat grains as well as contamination by the mycotoxins DON, 3-ADON, 15-ADON, and NIV. Samples were highly contaminated with type B trichothecenes, and 24% of the samples contaminated with DON were at concentrations higher than permitted by Brazilian legislation. Co-occurrence of these mycotoxins in oat grains samples was also observed; indicating the importance of further studies on trichothecene contamination in oat by-products as well as the toxic synergistic interactions of these mycotoxins to determine the potential risks to animal and human health.

## 5. Materials and Methods

### 5.1. Oat Samples

One hundred oat grain samples were collected from the states of Paraná and Rio Grande do Sul (50 samples from each region), the two largest oat-producing regions of Brazil. The grains were obtained after the cleaning and drying stages (up to maximum of 60 °C) of the 2018 harvest. Sampling was performed using a grain auger at different points of the harvest batches. At this stage, none of the grain samples appeared to have any visual fungal growth.

Each sample was homogenized and reduced to a subsample of 3 kg. Grains were transferred into polyethylene bags and kept at room temperature (for up to two days). The bags were then stored at −18 °C for mycotoxin analysis [46].

### 5.2. Water Activity and Identification of Mycobiota

Water activity analysis of the grain samples was conducted using the equipment Aqua-Lab CX-2, Decagon Devices. Samples were analyzed in triplicate. The serial dilution technique was used for fungal isolation [62]. Aliquots of each dilution were plated onto Dichloran Rose Bengal Chloramphenicol (DRBC, Oxoid) agar and incubated for 5 days at 25 °C, and the results are expressed in CFU/g.

Primary morphological characterization of the different genera was conducted according to [62], using Czapek yeast extract agar (CYA) and malt extract agar (MEA). Isolates belonging to the genus *Fusarium* were single-spored and plated onto potato dextrose agar (PDA) and carnation leaf agar (CLA) for further morphological characterization [63]. Isolates were stored in glycerol (60%) at −80 °C. For the FGSC strains, ma- croconidia formation was observed in CLA. PDA was used to verify the red pigment formed in the agar. *F. poae* was determined on CLA through visualization of the typical globose to napiform microconidia, formed in clusters on monophialides. In PDA, the mycelium color was pale to reddish-brown [63]

### 5.3. Characterization of Fusarium Isolates

The *Fusarium* isolates were initially identified as described above. Afterwards, 25 strains belonging to the *F. sambucinum* species complex (FGSC and *F. poae* isolates) were selected for sequencing and phylogenetic analyses. These isolates were selected in order to represent both regions studied (i.e., Paraná and Rio Grande do Sul). Sequencing reactions followed by phylogenetic analysis were performed on the *RPB2* locus [64,65,66].

Isolates were also characterized based on trichothecene genotyping (3-ADON, 15-ADON, and NIV) by multiplex PCR, following the methodology proposed in [67].

### 5.4. DNA Extraction, PCR, and Sequencing Analyses of the RPB2 Gene

*Fusarium* cultures were grown on PDA for 5 days at 25 °C, and the DNA was extracted using a DNeasy Plant Mini Kit (Qiagen, Hilden, Germany) according to the manufacturer’s instructions. PCR reactions and primer sets were performed according to [65,68]. PCR products were purified with QIAquick PCR Purification Kit (Qiagen, Hilden, Germany) and sequenced using Applied Biosystems^®^ 3500 Genetic Analyzer (Applied Biosystems, Foster City, CA, USA) by the company Helixxa Bases for Life (Paulínia, SP, Brazil).

The sequences were analyzed using Geneious v.6.0.6 (Biomatters, Auckland, New Zealand), and polymorphisms were confirmed by examining the chromatograms. For multiple alignment, nucleotide sequences were downloaded from the National Centre for Biotechnology Information (NCBI) and aligned with the obtained *Fusarium* oat isolate sequences using the ClustalW plugin in Geneious v.6.0.6 (Supplementary Materials Table S1).

### 5.5. Phylogenetic Analysis

Maximum parsimony analysis was performed using PAUP 4.0b10 (Sinauer Associates, Sunderland, MA, USA) [69]. A heuristic search option with 1000 random additional sequences and tree-bisection–reconnection algorithm for branch-swapping were used to infer the most parsimonious tree. Gaps were treated as missing data. The consistency index (CI) and retention index (RI) were calculated to verify the homoplasy present. Clade stability was verified through bootstrap analysis with 1000 replicates (PAUP 4.0b10), Bayesian inference analysis was also performed using the MrBayes plugin in Geneious v.6.0.6, run with a 2,000,000 generation Monte Carlo Markov chain method with a burn-in of 10,000 trees. *Fusarium concolor* was used as the outgroup. The phylogenies were visualized using FigTree v.1.4 (University of Edinburgh, Edinburgh, UK) [70].

### 5.6. Mycotoxin Analysis

#### 5.6.1. Mycotoxin Extraction

Mycotoxin extraction was conducted using QuEChERS, according to the manufacturer’s instructions. Initially, 300 g of oat grains were ground, and a subsequent subsample of 100 g was separated using a sieve (0.5 mm mesh 32, generating 0.5 mm particles) and homogenized. Then, 10 g of the ground sample was weighed and transferred into a 50 mL QuEChERS extraction tube, followed by 10 mL of ultrapure water and 10 mL of acetonitrile with 1.0% formic acid.

The sample was agitated vigorously for 1 min and then centrifuged for 5 min at 5000 rpm. After, 3 mL of supernatant was transferred to a 15 mL RoC QuEChERS centrifuge tube, containing 900 mg MgCl and 150 mg PSA (Primary and Secondary Amine Exchange Material—KS0-8924). This was shaken vigorously for 30 s and centrifuged for 5 min at 3700× *g* to separate the solid material. Finally, 1 mL of the supernatant was transferred into a flask for the solution to be evaporated in a heated sand bath at 60 °C.

Subsequently, the residue was diluted with 1 mL of acetonitrile:water (70:30 *v/v*), mixed and filtered through a 0.22 µm PTFE hydrophobic membrane filter and injected into a high-performance liquid chromatography with diode array detection.

#### 5.6.2. Chromatography Conditions

Chromatographic separation was performed through a high-performance liquid chromatograph (Shimadzu, Kyoto, Japan), Gemini C18 5.0 µm (250 × 4.6 mm) chromatographic column, and an auto-injector for injection handling of 20 µL, equipped with a diode-array detector SPD-M20A [71].

The mobile phase was composed of acetonitrile:water (70:30 *v/v*), with elution in the isocratic mode and a flow rate of 0.5 mL min^−1^, with a total analysis time of 15 min. The maximum absorption wavelength was 220 nm for 3-ADON, 15-ADON, DON, and NIV.

Data were collected and processed using LC Solution-Shimadzu software. The limit of detection (LOD), limit of quantification (LOQ), and recovery were: 16.15, 2.5, 2.5, and 16.15 µg/kg; 53.3, 8.3, 8.3, and 53.3 µg/kg; 98%, 92%, 84%, and 70% for DON, 3-ADON, 15-ADON, and NIV, respectively.

### 5.7. Statistical Analysis

Statistical analysis was performed using Statistix v.10 software. ANOVA and the Kruskal–Wallis test were chosen to assess the differences in *Fusarium* occurrence between the two studied regions as well as the differences in mycotoxin levels between the two studied regions. Values of *p* < 0.05 were considered statistically significant.

## Figures and Tables

**Table 1:** Frequency and mean count of fungal genera and *Fusarium* species complexes isolated from oat samples from two different regions of Brazil: Paraná (PR—50 samples) and Rio Grande do Sul (RS—50 samples).

Oat Origin	RS	PR	Average Count (CFU/g)
Oat a_w_ ^a^	0.54	0.51	
Genera of Fungi	Frequency (%)		
*Fusarium*	37.3	33.1	1.8 × 10^5^
*Phoma*	15.4	11.1	7.7 × 10^4^
*Epicoccum*	13.8	11.3	7.1 × 10^4^
*Alternaria*	9.6	16.3	5.9 × 10^4^
*Cladosporium*	7	6.9	3.3 × 10^4^
*Penicillium*	4.4	1.9	2.2 × 10^4^
*Aspergillus*	3.4	ND	2.3 × 10^4^
*Drechslera*	3	1.7	1.4 × 10^4^
*Pestalotiopsis*	1.5	0.3	4 × 10^3^
*Mucor*	1.5	1.7	7 × 10^3^
*Rhizopus*	1.5	2.6	9 × 10^3^
*Curvularia*	0.8	ND	6 × 10^3^
*Trichoderma*	0.8	ND	6 × 10^3^
*Nigrospora*	ND	13.1	4.3 × 10^4^
***Fusarium* Species Complexes**	**Frequency (%)**		
FSAMSC	93.8	85.5	1.7 × 10^5^
FTSC	3.2	0	7.6 × 10^3^
FFSC	3	6.7	4.3 × 10^4^
FIESC	ND	5	5.6 × 10^3^
FSSC	ND	2.8	3.1 × 10^3^

^a^ Mean value for water activity. ND: not detected; FSAMSC: *Fusarium sambucinum* species complex; FTSC: *F. tricinctum* species complex; FFSC: *F. fujikuroi* species complex; FIESC: *F. incarnatum-equiseti* species complex; FSSC: *F. solani* species complex.

**Table 2:** Occurrence of type B trichothecenes in oat grain samples from Rio Grande do Sul (RS) and Paraná (PR), Brazil.

Region	NIV			DON			15-ADON			3-ADON		
	Concentration (µg/kg)			Concentration (µg/kg)			Concentration (µg/kg)			Concentration (µg/kg)		
	Mean	Median	Range	Mean	Median	Range	Mean	Median	Range	Mean	Median	Range
PR	28.6	330.3	ND-820	45	540.1	ND-1620	7.7	349.7	ND-723.3	18.8	648.6	ND-2546.7
RS	46.7	778.3	ND-7716.7	35	503.2	ND-1610	3.6	157.8	ND-420	14.8	491.7	ND-3333.3

NIV: nivalenol; DON: deoxynivalenol; 15-ADON: 15-acetildeoxynivalenol; 3-ADON: 3-acetildeoxynivalenol; ND: not detected.

## Data Availability

Sequencing data are reported in Supplementary Table S1 and are available at the National Center for Biotechnology Information (NCBI) website (https://www.ncbi.nlm.nih.gov/, accessed on 23 November 2021), Accession Numbers: from MW929719 to MW929745.

## Supplementary Materials

The Supplementary Table S1 is available online at https://www.mdpi.com/article/10.3390/toxins13120855/s1. Table S1: *Fusarium* strains isolated from oats and reference taxa used for phylogenetic analysis.
